# A rare perspective: fine-needle aspiration cytologic features of hyaline vascular type of Castleman disease: A case report

**DOI:** 10.1097/MD.0000000000040972

**Published:** 2024-12-20

**Authors:** Xiang Shi, Yingyuan Wangsun, Meiping Wan, Sanyan Li

**Affiliations:** a Departments of Pathology, Qianjiang Central Hospital, Qianjiang, Hubei, China; b Nanchang University Jiangxi Medical College, Nanchang University, Nanchang, Jiangxi, China; c Department of Traditional Chinese Medicine, Qianjiang Central Hospital, Qianjiang, Hubei, China.

**Keywords:** cytopathology, fine needle aspiration cytology, hyaline vascular type of Castleman disease

## Abstract

**Rationale:**

Castleman disease, also known as Castleman syndrome, is a rare, nonmalignant lymphoproliferative disorder. The localized subtype of this disease is primarily the hyaline vascular type of Castleman disease (HVCD). Although this disease is a benign lesion, the histologic features are similar to those of some malignant lymphomas, so an accurate diagnosis of the disease is required. Lymph node hyperplasia lesions are usually diagnosed mainly by relying on biopsy histopathology, but the surgical process is time-consuming and expensive, and a part of patients usually refuse to undergo this examination method. Fine-needle aspiration cytology (FNAC) is a convenient, rapid, minimally invasive test for surface masses, and patients often receive cytopathology results in as little as a few minutes. Currently, there are few reports on the pathological features of FNAC in HVCD, so our cytopathological experience of using FNAC for rapid diagnosis of HVCD and other diseases that need to be differentiated are described in detail to give some meaningful references to the pathologists in recognizing HVCD from a rare cytological point of view.

**Patient concerns:**

A late 50s female patient presented to the hospital with a single subcutaneous enlarged nodule in the right side of the neck, which had been present for >6 months. The nodule exhibited a relatively clear border, and the patient presented with no signs of pain, no skin damage, and no other notable symptoms, but was concerned about the benignity or malignancy of the enlarged nodule.

**Diagnoses:**

To determine the exact diagnosis of the neck nodule, the patient first underwent FNAC, followed by surgical excision of the neck nodule for histopathologic examination.

**Interventions:**

Since HVCD is a benign lesion and the superficial neck nodes were removed by minimally invasive surgery, the patient did not receive any other interventions.

**Outcomes:**

We successfully and accurately diagnosed the rare HVCD using FNAC. Histologically similar lesion features of HVCD were successfully observed in cytology. The cytologic pattern of HVCD is distinctly different from that of other benign lymphoid tissue proliferative lesions or metastatic carcinomas.

**Lessons:**

This case shows important cytologic features for the diagnosis of HVCD using FNAC. The FNAC results showed abundant follicular dendritic cells and transparent blood vessels, and these cytologic features recapitulate the histopathologic alterations that were seen in HVCD. So by observing these cytologic features can help us to make an accurate diagnosis of HVCD using FNAC.

## 1. Introduction

Castleman disease (CD) is a rare lymphoproliferative disorder that was initially described by Dr Benjamin Castleman in 1956.^[1]^ This disease is heterogeneous and is typically classified into 2 major types; namely, unicentric Castleman disease (UCD) and multicentric Castleman disease (MCD).^[2,3]^ UCD often remains confined to a single lymph node or a group of lymph nodes, and patients may present with localized symptoms. UCD is often cured using surgical excision. By contrast,^[4]^ MCD affects multiple lymph nodes, and patients often present with complex systemic symptoms, such as fever, fatigue and weight loss, leading to challenges in treatment.^[5]^ CD predominantly manifests as UCD, while MCD is relatively rare.^[4]^

In terms of histopathology, CD commonly exhibits varying degrees of follicular center hyperplasia in lymph nodes, accompanied by regressive changes in the germinal centers and hyaline vascular blood vessels penetrating the atrophic germinal centers.^[6]^ Notably, these pathological changes are crucial for diagnosis. In the case reported in the present study, similar morphological characteristics were observed through fine-needle aspiration cytology (FNAC), suggesting the potential for diagnosing CD using this method. FNAC is a key examination method for the initial screening and diagnosis of superficial tumors in the human body.^[7]^ It is convenient, rapid and valuable in the diagnosis of numerous diseases. Therefore, the present study described the pathological diagnostic characteristics of hyaline vascular type of Castleman disease (HVCD) from the perspective of FNAC, which exhibits potential in the early diagnosis and treatment of the disease.

## 2. Case presentation

A late 50s female patient presented to hospital with a single subcutaneous enlarged nodule in the right side of the neck, which had been present for >6 months. The nodule exhibited a relatively clear border, and the patient presented with no signs of pain, no skin damage and no other notable symptoms. Notably, the patient had not undergone any treatment prior to visiting the hospital. Ultrasound examination indicated lymph node enlargement with homogeneous echoes and clear borders, the long axis of the abnormal lymph node is 1.39 cm, and the short axis is 0.51 cm. To further clarify the type of disease, the patient underwent FNAC using a 10-mL fine-needle syringe and a tissue pathology biopsy.

**Figure 1.**
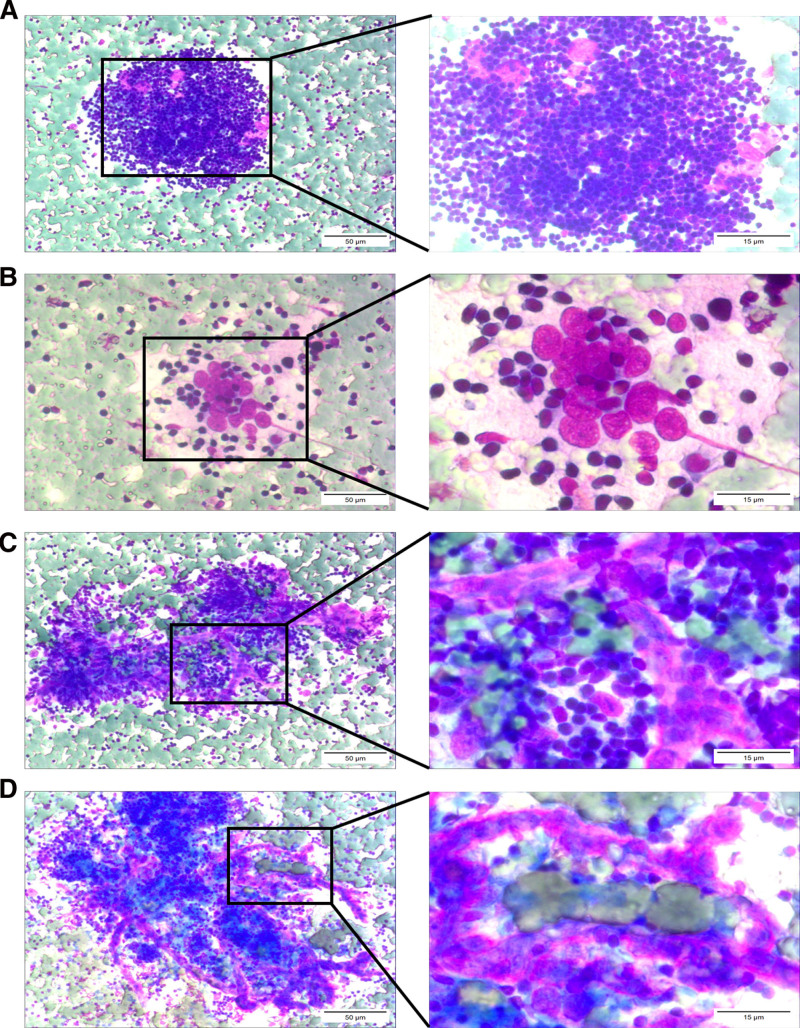
Cytopathological characteristics of HVCD (Liu’s ready-to-use staining). In fine-needle aspiration cytology, abundant lymph node follicular dendritic cells and scarlet small blood vessels can be observed. (A) Among a cluster of small lymphocytes, there are several large scarlet cells, which are follicular dendritic cells. (B) A cluster of follicular dendritic cells with bare nuclei, prominent nucleoli, and relatively large cell size. (C and D) Amidst a cluster of lymphocytes, there are numerous interspersed scarlet small blood vessels (scale bars, 50 μm; 15 μm in the magnified windows). HVCD = hyaline vascular type of Castleman disease.

Liu’s staining solution was used for FNAC. At room temperature, solution A was applied for 10 seconds, followed by the addition of solution B for a further 10 seconds, prior to rinsing in water. The sample was observed under a microscope (Olympus, Japan), and staining revealed a large number of dispersed small lymphocytes with fine chromatin. Numerous lymphocytes were clustered around scarlet cord-like hyaline vascular structures, which are the hyaline vessels extending into the germinal centers of the lymph nodes. Within these hyaline vessels, oval-shaped endothelial cells were aligned in the direction of the vessel extensions (Fig. 1). Notably, this vascular characteristic is a key cytological pathological feature of HVCD, and is an important differentiation factor from other lymph node lesions, including malignant lymphoma.

In addition, several large, aggregated oval-shaped scarlet cells with prominent nucleoli were observed among certain clusters of lymphocytes. These were labeled as follicular dendritic cells (FDCs) within the atrophied germinal centers. Notably, these exhibit atrophy of the germinal centers, compared with healthy lymph nodes (Fig. 1). FDCs are a type of dendritic cell located in the follicles of lymph nodes. They play a crucial role in humoral immune responses through capturing and presenting antigens, to activate and maintain lymphocyte responses.^[8]^

**Figure 2.**
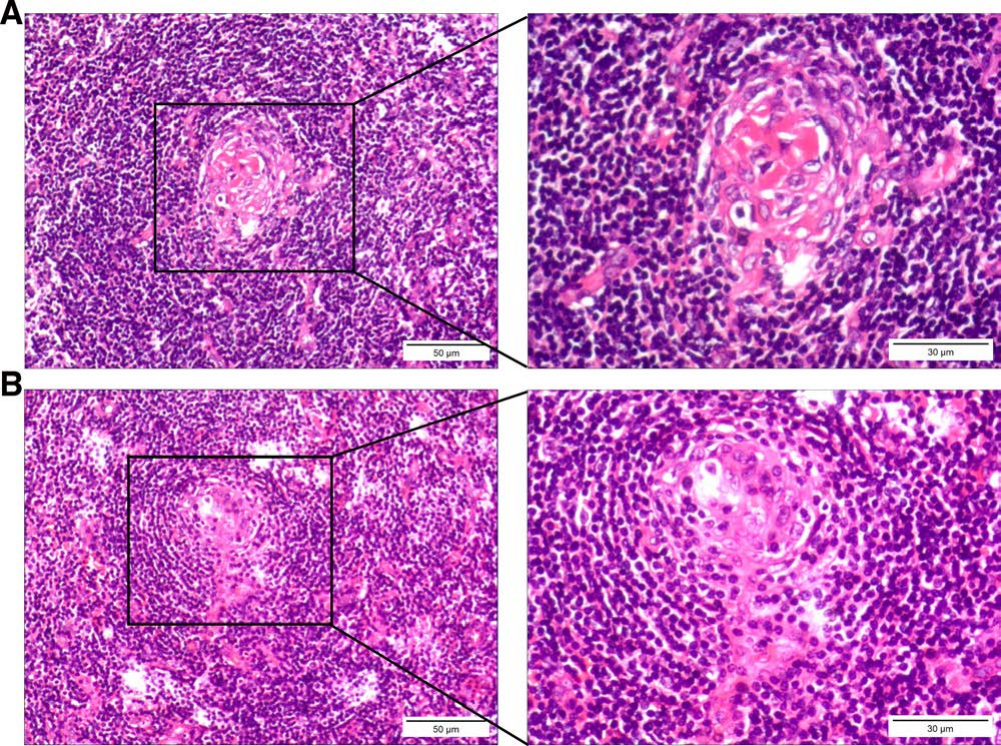
Histopathological characteristics of HVCD (hematoxylin and eosin staining). HVCD often manifests as varying degrees of hyperplasia in the follicular centers of lymph nodes, accompanied by degenerative changes in the germinal centers, hyalinized blood vessels extend into the atrophic germinal centers. (A) Large, prominent follicular dendritic cells are present within the atrophic germinal centers. (B) The mantle zone cells surrounding the atrophic germinal center are arranged in a concentric pattern, and a distinct small blood vessel can be seen extending into the germinal center (scale bars, 50 μm; 30 μm in the magnified windows). HVCD = hyaline vascular type of Castleman disease.

Subsequently, H&E staining was performed in the present study. In brief, paraffin-embedded sections (thickness, 4 μm) were treated with hematoxylin reagent at 37°C for 5 minutes, followed by incubation in a hematoxylin reagent box for a further 5 minutes. Sections were subsequently treated with 1% hydrochloric acid-ethanol solution for 1 second. Following treatment with hydrochloric acid-ethanol solution, sections were stained with eosin at 37°C for 3 minutes. Sections were dehydrated and mounted, and images were obtained using an optical microscope. Pathological examination of tissue biopsy specimens revealed hyperplastic lymph nodes, with atrophic germinal centers that primarily contained FDCs and vascular endothelial cells. The mantle zones were widened and arranged in a concentric pattern, while the interfollicular areas were vascular-rich, extending into the atrophic germinal centers of the lymph nodes (Fig. 2). These are key morphological features indicative of HVCD.

## 3. Discussion

CD is a rare non-neoplastic proliferative disorder of lymphoid tissue that may occur at any age.^[9]^ Based on the severity of the disease, it is mainly classified into 2 types; namely, UCD and MCD.^[10]^ Pathologically, CD is further divided into HV and plasma cell types.^[10]^ UCD is localized and often presents as the HV type, and is the most common type of CD. Patients with UCD often present with localized lymph node enlargement and no notable symptoms.^[11]^

**Figure 3.**
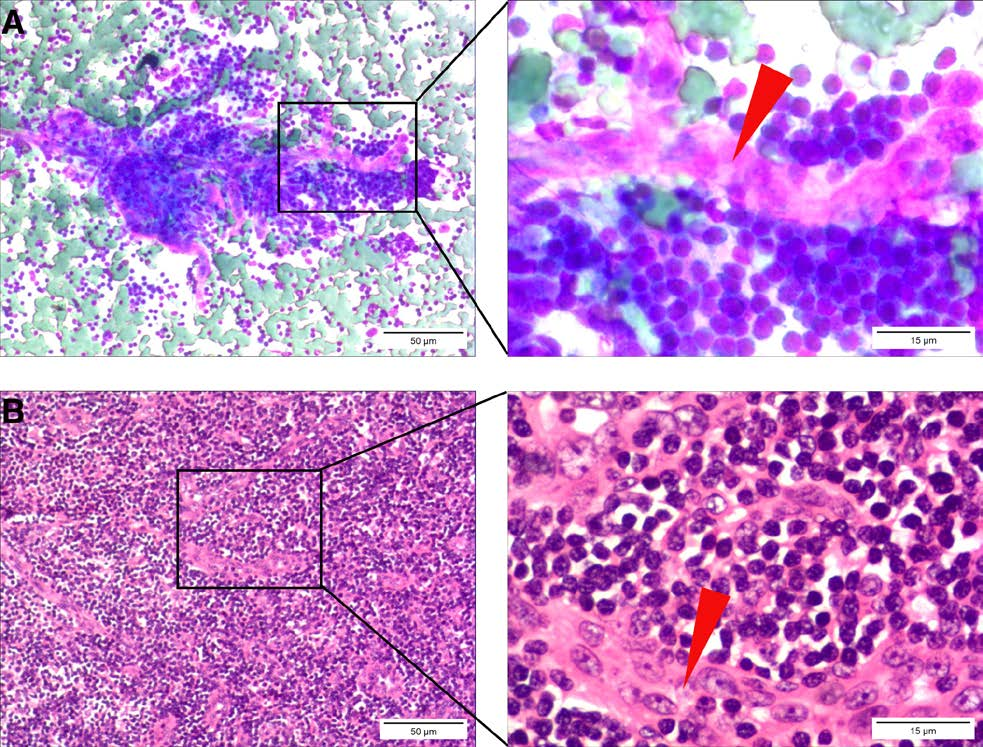
The morphological comparison of proliferating small blood vessels in cytology and histopathology. (A) In fine-needle aspiration cytology, scarlet translucent blood vessels are observed interspersed among lymphocytes. (B) In histopathology, abundant small blood vessels seen among lymphocytes exhibit similar morphology and distribution to those in cytology. The red arrow points to the proliferative small blood vessels (scale bars, 50 μm; 15 μm in the magnified windows).

**Figure 4.**
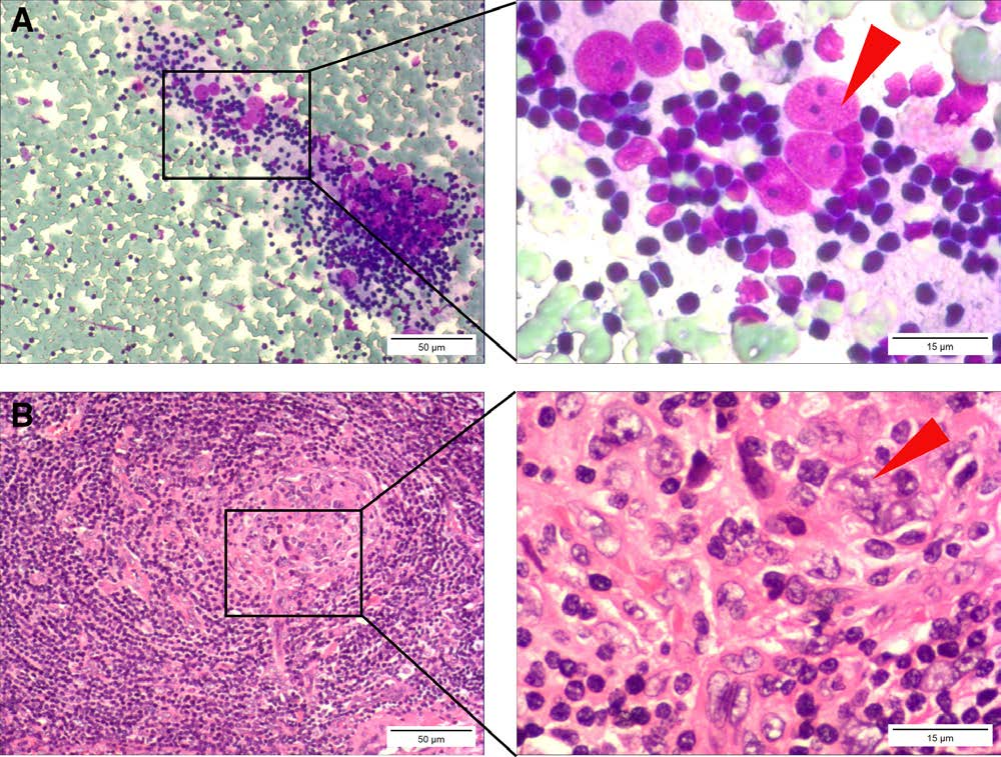
The morphological comparison of follicular dendritic cells in cytology and histopathology of lymph nodes. (A) In fine-needle aspiration cytology, follicular dendritic cells appear larger in size, with prominent nucleoli, regular cells, and bare nucleus-like morphology. (B) In histology, follicular dendritic cells also appear larger in size, with distinct nucleoli, similar to the morphology observed in cytology. The red arrow points to follicular dendritic cells (scale bars, 50 μm; 15 μm in the magnified windows).

The histopathological characteristics of CD are diverse; however, this disease often presents with distinct recognizable features.^[12]^ HVCD manifests as the regressive transformation of the lymph node germinal centers, with widened mantle zones arranged in concentric patterns and interfollicular areas rich in venules. In addition, this type of CD exhibits venules that extend into the regressed germinal centers of the lymph nodes.^[12,13]^ In the present case, scarlet venules penetrating between aggregates of lymphocytes, with abundant FDCs and vascular endothelial cells, were observed using FNAC. Notably, the observed cytological characteristics corresponded with the observed histopathological features of HVCD, leading to the cytological diagnosis of HVCD (Figs. 3 and 4).

**Figure 5.**
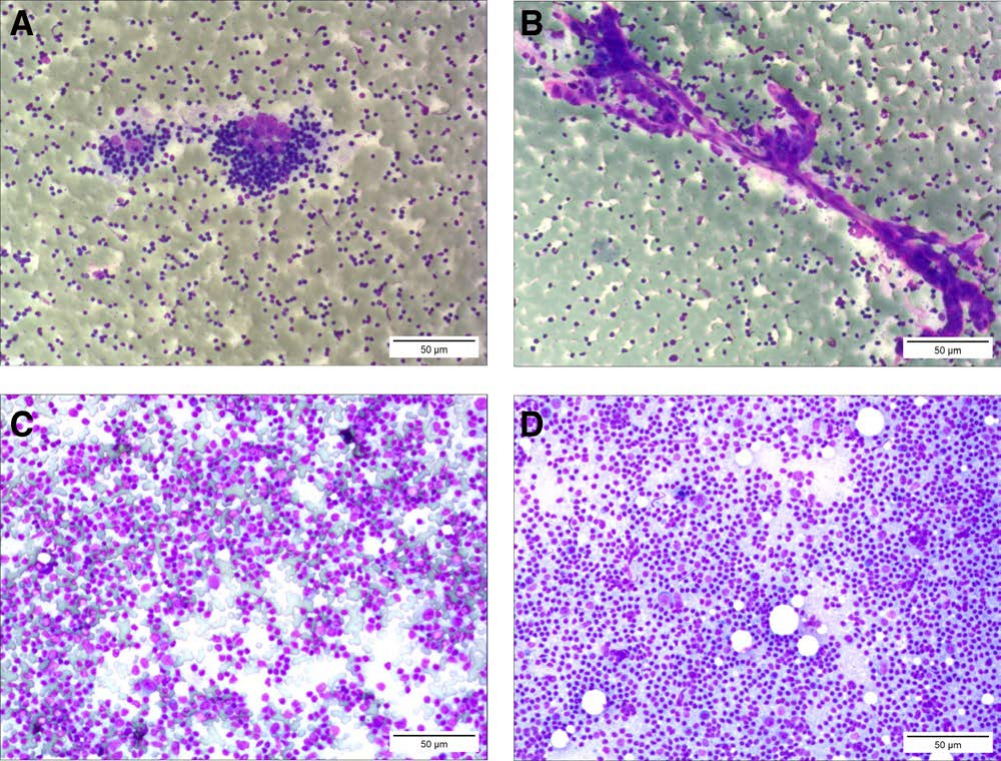
Comparison of the morphology in fine-needle aspiration cytology between HVCD and benign lymph node reactive hyperplasia. (A) Small clusters of follicular dendritic cells in HVCD. (B) The proliferated small blood vessels in HVCD. (C and D) In benign reactive lymphoid hyperplasia, there is a rich mixture of large lymphocytes and small lymphocytes with regular nuclei. The abundant follicular dendritic cells and small blood vessels seen in HVCD are not easily observed (scale bars, 50 μm). HVCD = hyaline vascular type of Castleman disease.

**Figure 6.**
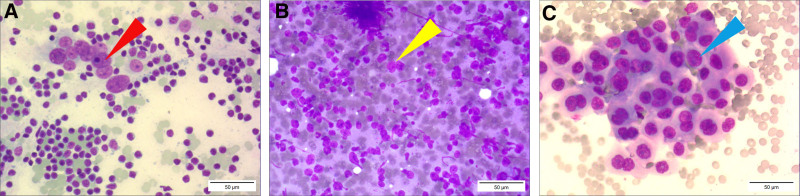
A morphological comparison of HVCD, malignant lymphoma, and metastatic cancer in fine-needle aspiration cytology. (A) Follicular dendritic cells in HVCD, its characteristics include a relatively large cell size, regular nuclei, and 1 or 2 prominent nucleoli, making it easily confusable with the latter 2 types of cells. The red arrow points to follicular dendritic cells. (B) Malignant lymphoma cells exhibit clonal proliferation, with a diffuse and uniform cell size, irregular nuclei, and nucleoli that are not as prominent as those in follicular dendritic cells. The yellow arrow points to malignant lymphoma cells. (C) Metastatic carcinoma in the lymph node shows clustered tumor cells with irregular nuclei, increased nucleus-to-cytoplasm ratio, and prominent nucleoli. The blue arrow points to metastatic carcinoma cells (scale bars, 50 μm). HVCD = hyaline vascular type of Castleman disease.

In previous studies, FNAC revealed a mixed population of abundant large and small lymphocytes with regular cellular morphology, indicative of benign reactive lymphoid hyperplasia.^[14,15]^ This disease rarely exhibits clusters of FDCs or abundant hyaline vascular structures, differentiating it from HVCD (Fig. 5). In addition, the diffuse presence of monoclonal lymphocytes with irregular cellular morphology is indicative of malignant lymphoma, and this was observed using FNAC.^[16]^ By contrast, HVCF demonstrated nonclonal, predominantly small lymphocytes, accompanied by abundant blood vessels and FDCs. Notably, these morphological features are absent in malignant lymphoma; thus, differentiating this disease from others. Metastatic cancer cells in lymph nodes often exhibit highly irregular large nuclei with an increased nucleocytoplasmic ratio.^[17]^ Although FDCs possess large nuclei in HVCD, these cells lack atypical changes, and present as naked nuclei (Fig. 6). However, lymphoid disorders are complex, and differentiating between various lymphatic diseases requires a high degree of correspondence between histopathological and molecular diagnostic results.^[18]^

Although the present study may provide novel insights into the cytology of HVCD, there are limitations. For example, due to the rarity of CD, the sample size was small, which may affect the generalizability of the results. Thus, further analyses are required using additional cases. Moreover, CD is divided into 2 types as previously described, and the plasma cell type was not described in the present study.^[19]^ Thus, the present study did not differentiate the cytological characteristics between both types. FNAC has no explicit contraindications and can be performed on any palpable masses accessible on the body surface, with results available quite rapidly. However, the diagnostic accuracy of FNAC is not as high as that of tissue biopsy. For diseases with distinct cytological features, FNAC can provide an accurate diagnosis. Conversely, for diseases with nonspecific cytological morphology, it cannot directly identify the specific disease but can only offer guesses about the type of disease or a preliminary assessment of whether it is benign or malignant. It has been reported that abnormal expression of IL-6 is present in MCD, and HHV-8 infection is often observed in HIV-associated MCD.^[20]^ Overexpression of VEGF is also an important molecular feature in CD.^[21]^ However, due to the limited number of studied cases, these changes cannot be definitively confirmed.

## 4. Conclusion

In conclusion, a patient was diagnosed with HVCD using FNAC and histopathology. The present study revealed the typical pathological characteristics of HVCD using FNAC, and highlighted the diagnostic value of this method. The present study may provide a novel theoretical basis for the use of FNAC in the diagnosis of HVCD, which may lead to the early diagnosis and treatment of this disease.

## Author contributions

**Data curation:** Xiang Shi, Yingyuan Wangsun, Meiping Wan, Sanyan Li.

**Investigation:** Sanyan Li.

**Project administration:** Xiang Shi, Yingyuan Wangsun, Meiping Wan, Sanyan Li.

**Resources:** Xiang Shi, Yingyuan Wangsun, Meiping Wan.

**Software:** Xiang Shi.

**Writing – original draft:** Xiang Shi, Yingyuan Wangsun, Meiping Wan, Sanyan Li.

**Writing – review & editing:** Xiang Shi, Yingyuan Wangsun, Meiping Wan, Sanyan Li.
